# Recovery after brain injury: mechanisms and principles

**DOI:** 10.3389/fnhum.2013.00887

**Published:** 2013-12-24

**Authors:** Randolph J. Nudo

**Affiliations:** Department of Molecular and Integrative Physiology, Landon Center on Aging, University of Kansas Medical CenterKansas, KS, USA

**Keywords:** motor cortex, stroke, traumatic brain injury, axonal sprouting, motor learning, recovery

## Abstract

The past 20 years have represented an important period in the development of principles underlying neuroplasticity, especially as they apply to recovery from neurological injury. It is now generally accepted that acquired brain injuries, such as occur in stroke or trauma, initiate a cascade of regenerative events that last for at least several weeks, if not months. Many investigators have pointed out striking parallels between post-injury plasticity and the molecular and cellular events that take place during normal brain development. As evidence for the principles and mechanisms underlying post-injury neuroplasticity has been gleaned from both animal models and human populations, novel approaches to therapeutic intervention have been proposed. One important theme has persisted as the sophistication of clinicians and scientists in their knowledge of neuroplasticity mechanisms has grown: behavioral experience is the most potent modulator of brain plasticity. While there is substantial evidence for this principle in normal, healthy brains, the injured brain is particularly malleable. Based on the quantity and quality of motor experience, the brain can be reshaped after injury in either adaptive or maladaptive ways. This paper reviews selected studies that have demonstrated the neurophysiological and neuroanatomical changes that are triggered by motor experience, by injury, and the interaction of these processes. In addition, recent studies using new and elegant techniques are providing novel perspectives on the events that take place in the injured brain, providing a real-time window into post-injury plasticity. These new approaches are likely to accelerate the pace of basic research, and provide a wealth of opportunities to translate basic principles into therapeutic methodologies.

## Introduction

After injury to the cerebral cortex, as often occurs in stroke or traumatic brain injury (TBI), a large portion of the forebrain sensory-motor apparatus is affected, including the frontal and parietal cortex and/or subcortical structures in the striatum and thalamus, resulting in deficits in motor function in the contralateral musculature. However, substantial spontaneous recovery occurs in the weeks to months following injury. Understanding how the remaining sensory-motor structures can support the recovery of such functions has been a primary goal of recent neuroscientific research. This paper will review the current theoretical models and empirical evidence for functional plasticity in the cortical motor system. These data provide a basic understanding of plasticity principles needed to understand and optimize the effects of therapeutic interventions designed to promote adaptive plasticity.

## Mechanisms underlying experience-dependent plasticity in motor cortex

Decades of experimentation in the cerebral cortex have demonstrated many physiological and anatomical examples of cortical plasticity. While such phenomena have now been observed in widespread cortical areas, the present article will focus exclusively on somatosensory and motor cortex, due to their importance in understanding motor recovery after brain injuries. Though these processes are triggered by several endogenous and exogenous events, one of the most potent modulators of cortical structure and function is behavioral experience (Nudo et al., 1996a; Karni et al., 1998; Kleim et al., 1998). Emergent properties of each cortical area are shaped by behavioral demands, driven largely by repetition, and temporal coincidence. For example, skilled motor activities requiring precise temporal coordination of muscles and joints must be practiced repeatedly. Such repetition is thought to drive the formation of discrete modules where the conjoint activity is represented as a unit.

Clues to understanding plasticity in adult brains can be found throughout the developmental neuroscience literature. During brain development, guidance cues for axonal sprouting are activity-dependent. There are two phases in the maturation of thalamocortical connections. In the first phase, thalamocortical axons are directed to their cortical targets by axonal guidance molecules. This process may involve spontaneous neural activity. In the second phase, cortical activity guides axonal sprouting within the cerebral cortex, determining topological connectivity patterns. Postnatal axonal branching patterns within cerebral cortex have also been shown to involve sensory related stimulus activity possibly by initiating molecular retrograde signals such as BDNF (Uesaka et al., 2006).

Though long-range axonal sprouting was once thought to be non-existent in adult animals, injury creates a particularly ripe environment for axonal sprouting processes to be re-initiated. After a focal ischemic infarct in rats, synchronous neuronal activity is a signal for post-infarct axonal sprouting to be initiated from the intact cortical hemisphere to peri-infarct cortex and the contralateral dorsal striatum (Carmichael and Chesselet, 2002). Thus, evidence now supports the importance of cortical activity for axonal sprouting within the developing and adult brain. It follows that differences in post-infarct behavioral experience may influence which neurons become targets for both local and distant sprouting axons by differentially activating task-specific cortical areas.

It is important to point out that context-dependent reinforcement is critical for such plasticity to occur in cortical neurons of adult animals. That is, simple exposure to sensory stimuli causes little or no long-lasting change in receptive field properties. This principle was illustrated in a set of studies in which both somatosensory and auditory stimuli were presented to animals. The animals were rewarded for discriminating physical properties of only one of the modalities. Receptive field plasticity was seen in the cortex corresponding to the relevant sensory modality, but not the irrelevant modality (Recanzone et al., 1992).

**Figure 1:**
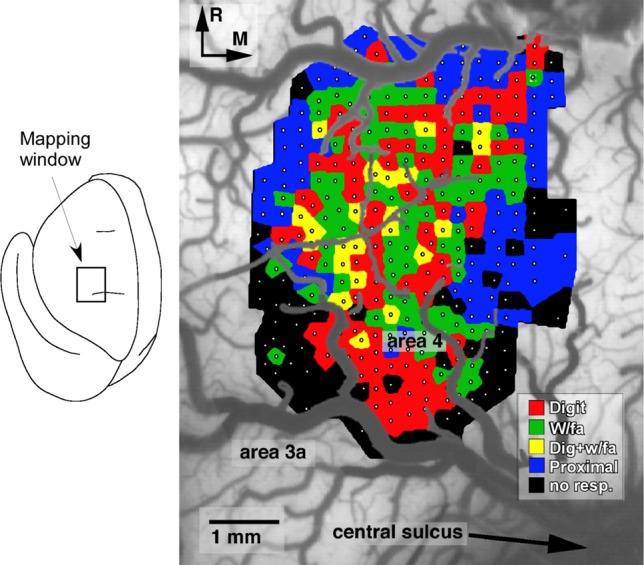
**Representation of distal forelimb movements in primary motor cortex (area 4) of a squirrel monkey**. Under ketamine sedation, movements were evoked by intracortical microstimulation at each of 321 sites (small white dots) located approximately 250 μm apart. The distal forelimb representation is comprised of digit (red), wrist (w/fa; green), forearm (green) movements, as well as combinations of single-joint movements (yellow). This fractionated pattern of movement representations is due to the intermingling of corticospinal neurons that project to different subsets of motor neurons (Milliken et al., 2013).

Several general principles of motor map organization have been demonstrated that are thought to underlie the ability of the motor cortex ability to encode motor skills (Monfils et al., 2005). First, motor maps are fractionated, in that they contain multiple, overlapping representations of movements (Figure 1). Second, adjacent areas within cortical motor maps are highly interconnected via a dense network of intracortical fibers. Third, these maps are extremely dynamic and can be modulated by a number of intrinsic and extrinsic stimuli. Together, these characteristics provide a framework that facilitates the acquisition of novel muscle synergies, at least in part, through changes in the intracortical connectivity of individual movement representations (Capaday et al., 2013).

However, the dynamic nature of motor maps belies the issue of stable neural connections that must be maintained to respond to environmental demands and retain acquired motor skills. In fact, using stimulus-triggered averaging of electromyographic activity in M1 of macaque monkeys, it appears that facilitation and suppression of individual muscles are surprisingly stable despite alterations in joint-angles, postural changes and various phases of a task (Griffin et al., 2009). Within the cortex, this balance is thought to be achieved through interactions of the excitatory and inhibitory connections of pyramidal cells and local inhibitory networks (Huntley and Jones, 1991; Aroniadou and Keller, 1993). This in turn requires an internal mechanism that is capable of shifting this balance toward strengthening relevant synaptic connections.

Horizontal fiber connections have been shown to arise from excitatory pyramidal neurons and allow for the co-activation of adjacent and non-adjacent cortical columns. In addition to activating excitatory pyramidal cells, they also generate inhibitory responses via the activation of GABAergic interneurons (Jones, 1993). Furthermore, the activity of these horizontal fibers has been shown to be mediated by both long-term potentiation (LTP) and long-term depression (LTD) between distant motor cortical areas (Hess et al., 1996). In slice preparations after motor learning, rats have larger amplitude field potentials in the motor cortex contralateral to the trained forelimb (Rioult-Pedotti et al., 1998). Thus, the synaptic strength of horizontal connections in the motor cortex is modifiable and may provide a substrate for altering the topography of motor maps during acquisition of motor skills. Together, these horizontal fiber characteristics provide a mechanism capable of both facilitating the activation of multiple novel muscle synergies that are required for motor skill acquisition, while likewise providing a mechanism, via inhibitory processes, of motor map stability that is required to maintain stable, neural representations in response to irrelevant (i.e., untrained) environmental events.

The hypothesis that Hebbian-like changes in intracortical synaptic connections link different cortical neurons to form functional modules gained further support by a study in the motor cortex of adult macaque monkeys. This study demonstrated that the output properties of motor cortex neurons can be altered by artificially coupling neuronal discharge patterns (Jackson et al., 2006). Electrodes were implanted in motor cortex of monkeys and two sites were selected on the basis of their response to ICMS. ICMS produced different movements at the two sites, which were located 1–2 mm apart. Then spike discharges were recorded from one site (Site 1) and used to stimulate the second site (Site 2) with a predetermined delay. When ICMS was used to determine the output properties of the two sites a few weeks later, it was found that Site 2 acquired the properties of Site 1—the ICMS-evoked movements were identical. This study provides further support for the notion that temporal correlation of inputs and outputs drives the emergence of coupling among motor cortex modules.

Structural alterations also occur in adult animals as a consequence of experience. Dendritic and synaptic morphology of motor cortex neurons are altered by specific motor learning tasks (Jones et al., 1999; Kleim et al., 2002b). Both LTP-like processes and dendritic spine expansion has been demonstrated in the same preparation in the motor cortex during a skilled learning task (Harms et al., 2008). Dendritic spine formation occurs quite rapidly, within 1 h on pyramidal neurons in the motor cortex contralateral to the limb performing the task. Further, subsequent training stabilizes the expanded spines, presumably as a basis for long-term memory of skilled motor tasks (Xu et al., 2009). Structural changes are highly specific to the neurons relevant for the skilled task (Wang et al., 2011). Classes of genes associated with early phases of motor skill learning have been identified and include those known to regulate synaptic plasticity, synaptogenesis, and cytoskeletal dynamics (Cheung et al., 2013). However, it would appear that widespread synaptogenesis and motor map plasticity are only evident in relatively late phases of motor skill acquisition, when motor memories for the skill are well-established (Kleim et al., 2004; Xu et al., 2009).

Dopamine appears to play a significant role in acquisition of skilled motor tasks (Hosp and Luft, 2013). Motor cortex, at least in rat, receives a substantial number of dopamine terminals, most from the ventral tegmental area. Luft and colleagues found that after dopamine depletion (by injection of 6-hydroxy-dopamine), rats are impaired at learning a pellet retrieval task. However, if they had already learned the task, the performance was not impaired. The impairment is reversed by injection of levodopa. These and other studies by this group provide strong evidence that dopamine specifically plays a role in motor memory consolidation.

## Motor skill learning and plasticity in motor maps

The term “motor learning” is not rigidly defined in most experimental models, but instead thought of as a form of procedural learning that encompasses such elements as skill acquisition and motor adaptation. More specific is motor skill learning itself, which is often described as the modification of the temporal and spatial organization of muscle synergies, which result in smooth, accurate, and consistent movement sequences (Hammond, 2002). Paralleling animal experiments, functional magnetic imaging studies in humans have led to the hypothesis that motor learning is a two stage process (Ungerleider et al., 2002). The first stage is rapid, and results in within-session decreases in neural activity. The second, slower stage results in increases and expansion of activity in M1.

One technique that has provided valuable information regarding functional plasticity in motor maps in experimental animals is intracortical microstimulation (ICMS) (Figure 1). As employed in plasticity studies in motor cortex of non-human primates, the ICMS protocol utilizes glass microeletrodes (10–20 μm tips) filled with 3.5 M NaCl. Current is delivered through a platinum wire inserted in the stimulating electrode. The stimulus consists of 13, 200μs cathodal, monophasic pulses delivered at 350 Hz, with a maximum current of 30μA. The electrode is lowered perpendicular to the surface of the cortex to a depth of 1750 μm, which targets layer V of the cortex, the location of the corticospinal cell bodies. Current levels required for evoking overt movements in anesthetized animals are lowest at this depth. Electrode penetrations are made at 250 μm increments, and recorded on a digital picture of the cortical surface. This procedure allows for the derivation of high resolution maps of motor cortical movement representations with negligible damage to the tissue, thus allowing for repeated mapping procedures within the same subject. Typically, baseline maps are derived prior to behavioral training. These maps consist of digit and wrist/distal forelimb movement representations, bordered medially, rostrally, and laterally by proximal shoulder representations. The caudal border is composed of somatosensory cortex (area 3a), and thus movements are rarely evoked at the current intensities employed in these studies (max of 30μA).

Based on ICMS results in non-human primates, the general topographic representation of specific body parts is quite consistent in M1, but substantial individual variability exists in the detailed topography on a more refined level, e.g., within the hand representation. The size of the hand representation can vary by over 100% in different monkeys, a difference that cannot be accounted for on the basis of the animal's size alone. It has been hypothesized that individual variation in motor maps is a result of each individual's sensorimotor experiences leading up to the motor mapping procedure (Nudo et al., 1992).

To examine the relationship between motor skill learning and changes in motor map representations, a manual dexterity task is often utilized. The typical apparatus consists of a plexiglas board that is attached to the front of the monkeys' home cage. The board contains food wells of different sizes: the largest is large enough to insert the entire hand, while only one or two digits can be inserted into the smallest well. Small, flavored food pellets are placed in the wells one at a time. While initial performance on the task is typically very poor, the monkeys are quite adept and came become very skilled on the task, retrieving 500–600 pellets per day within 1–2 weeks.

**Figure 2:**
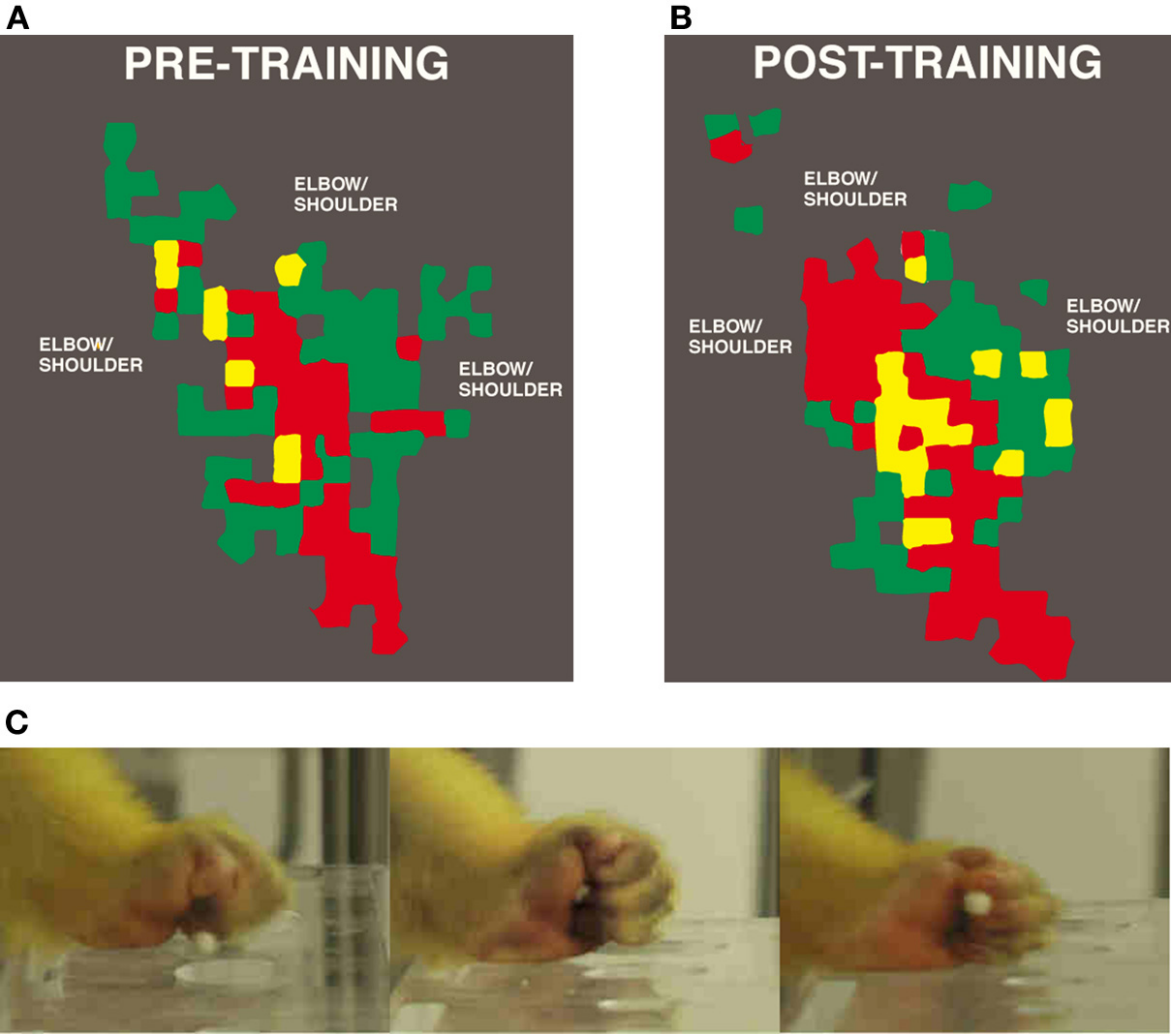
**Representation of distal forelimb representations in motor cortex after digit skill training as defined by intracortical microstimulation**. Digit areas (red) expand after only 12 days of training. Combination movements that reflect the individual kinematics that the monkey employs also expand their representations. **(A)** Pre-training map. **(B)** Post-training map. **(C)** Still images of squirrel monkey retrieving food pellets from small wells (Nudo et al., 1996a).

Utilizing manual dexterity training in combination with ICMS maps has been crucial in demonstrating the dynamic relationship between motor skill learning and cortical map plasticity. The first study to directly examine this relationship used varying behaviorally demanding tasks to selectively activate specific components of motor maps (Nudo et al., 1996a). This study used three monkeys trained on the manual dexterity task to retrieve food pellets using primarily using digit and wrist movements. A fourth subject was trained to use its wrist and forearm to receive pellets by turning a rotatable eye-bolt. Training continued for approximately 10–11 days, sufficient for an asymptotic level of performance to be reached. Post-training ICMS mapping revealed changes in motor map topography that directly reflected the demands of the particular behavioral task. Thus, monkeys trained on the manual dexterity task showed an increase in digit representations and corresponding reduction in wrist and shoulder representations compared to pre-training maps (Figure 2), while the monkey trained to turn the eye bolt exhibited the opposite effects—an increase in wrist and forearm representations at the expense of digit representations.

In addition, an increase in ICMS-evoked multi-joint movements was observed. These movements consisted of simultaneous executions of digit and wrist or proximal movements at low ICMS current levels, and were only observed after training on the digit-use intensive manual dexterity task. Both before and after training, thresholds for evoking multi-joint responses were significantly lower than single joint responses. These results imply that behaviorally relevant, simultaneous or sequential movements may become associated in the motor cortex through repeated activation. It is possible that temporal correlation of inputs and outputs in the motor cortex drives emergent properties, as it seems to do in somatosensory cortex. Thus, muscle and joint synergies used in complex, skilled motor actions may be supported by alterations in local networks within the motor cortex. As skilled tasks become more stereotyped in timing of sequential joint movements, functional modules emerge in the cortex to link the outputs of different motoneuron pools.

## Motor skill learning vs. motor use

These findings lead to the question of what aspects of motor skill learning drive the observed changes in map representations. Given that in the previous experiments, subjects were trained repeatedly on the same motor skills task, it is possible that increased muscle activity alone produced the observed changes in map representations. To address this issue, a group of monkeys was trained exclusively on either the largest or the smallest well in the digital dexterity task. The rationale in this design is that the largest well allows for simple multi-digit movements for pellet retrieval, which does not require the subject to develop novel skilled digit movements, since simply grasping for food is a normal part of their daily home cage behavior, and this is already part of their behavioral repertoire. Small well food pellet retrieval, in contrast, requires the monkey to manipulate 1–2 digits to retrieve the pellet, which is considerably harder given that squirrel monkeys lack monosynaptic corticospinal projections to motoneurons, which probably limits individuation of digit movements (Lemon and Griffiths, 2005).

**Figure 3:**
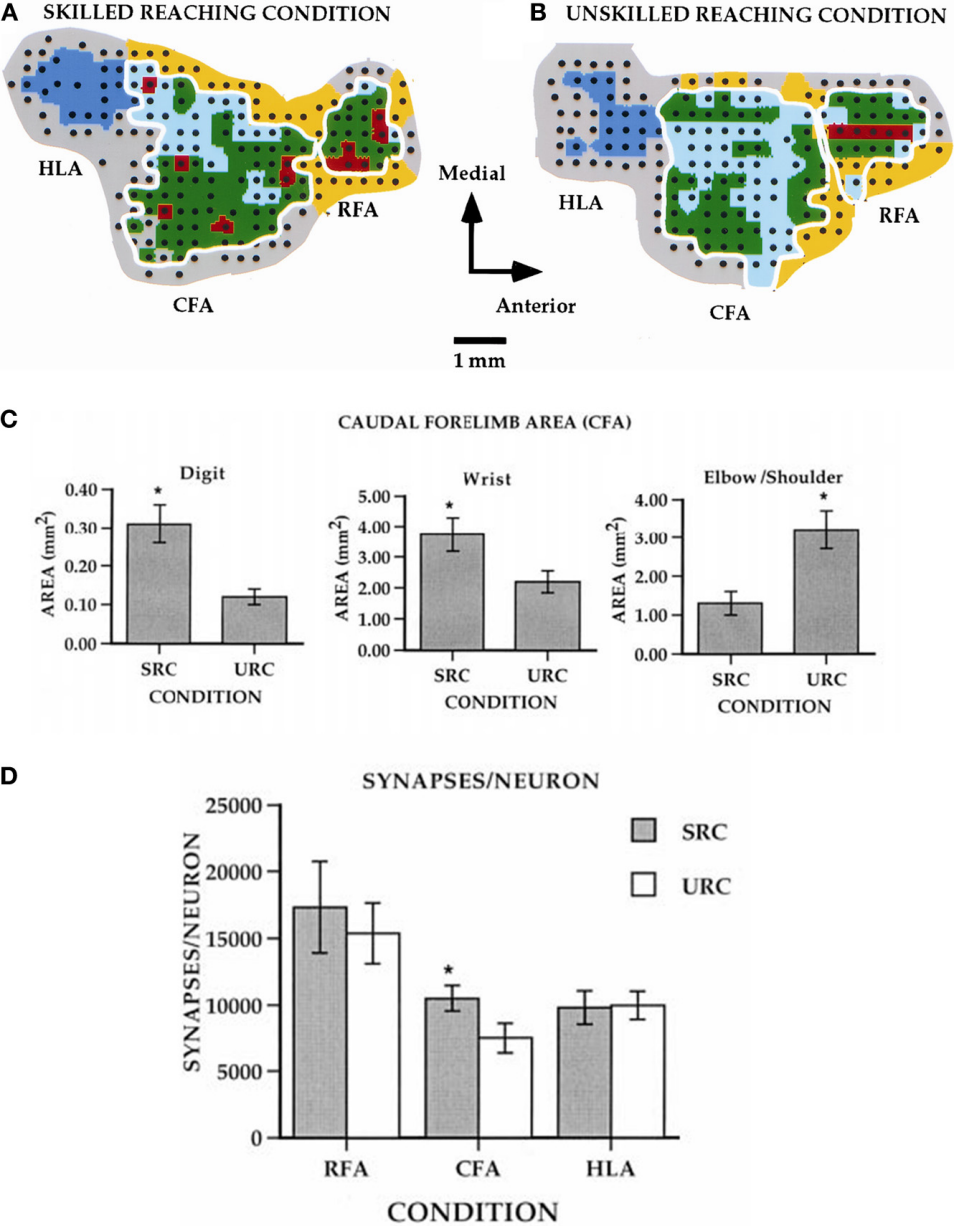
**Differential effects of skill vs. use. (A)** ICMS-derived motor map (digit, red; wrist, green; elbow/shoulder, light blue) of a rat that learned a skilled reaching movement. **(B)** ICMS-derived motor map of a rat that learned to press a bar. The two forelimb areas are outlined in white. The caudal forelimb area (CFA) is separated from the rostral forelimb area (RFA) by a band of head/neck representations (yellow). The hindlimb area (HLA) is shown in dark blue and nonresponsive sites in gray. **(C)** Note the enlarged digit and wrist/forearm representations in the skilled reaching condition (SRC), and enlarged should representation in the unskilled reaching condition (URC, bar press). **(D)** In the CFA, synapses per neuron were significantly increased (^*^p < 0.05), but no changes occurred in RFA or HLA (Kleim et al., 2002a).

Compared to pre-training maps, monkeys trained on the large well pellet retrieval did not show an expansion of the digit representation, while those trained on the small well did exhibit an expansion of the digit representation (Plautz et al., 2000). These findings strongly suggest that an increase in motor activity in the absence of motor skill acquisition is insufficient to drive neurophysiological changes in the motor cortex. Similar findings have been found in rodents examining pellet retrieval vs. bar pressing. Rats that learned to retrieve pellets from a rotating platform displayed more distal movements in their motor maps. This expansion was associated with significant synaptogenesis (Kleim et al., 1998, 2002a). Rats that simply pressed a bar showed no map changes or synaptogenesis (Figure 3). Thus, plasticity in motor cortex can be said to be skill- or learning-dependent, rather than strictly use-dependent. Tasks that require acquisition of new motor skill induce neurophysiologic and neuroanatomic changes in motor cortex, but simple repetitive motion or strength training tasks do not (Kleim et al., 1998; Plautz et al., 2000; Remple et al., 2001).

## Injury-induced plasticity in motor cortex

Deficits in motor function are common in numerous neurological conditions. However, the adult central nervous system retains an impressive capacity to recover and adapt following injury. Such so-called spontaneous (or natural) recovery occurs after spinal cord injury, TBI, and stroke. Therefore, a basic understanding of the mechanisms that underlie spontaneous recovery of function is the initial step in the development of modulatory therapies that may improve recovery rates and endpoints. While injury confined to one or another motor field occurs only in certain middle cerebral artery (MCA) strokes and focal TBI or neurosurgical resections, experimental data from animal models is extremely valuable in identifying the mechanisms underlying motor recovery after CNS injury.

**Figure 4:**
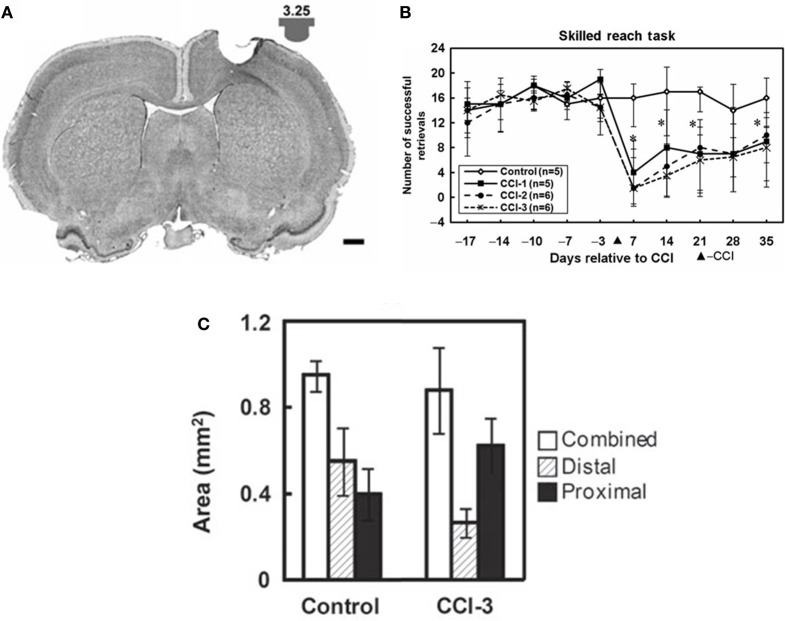
**Reorganization of the rat premotor cortex after controlled cortical impact in the primary motor cortex. (A)** Coronal section through the primary motor cortex (caudal forelimb area, or CFA) of a rat approximately one month after a controlled cortical impact. Impactor tip dimension and shape is shown in the inset. **(B)** Behavioral performance on a single-pellet retrieval task before and after the injury (^*^p < 0.05). **(C)** Alteration in motor maps in the rat premotor cortex (rostral forelimb area, or RFA) approximately one month after a controlled cortical impact. In the premotor area that was spared by the lesion, digit representations contracted, while proximal representations expanded. This suggests that the behavioral recovery that was observed was due to compensatory kinematic patterns rather than true recovery (Nishibe et al., 2010).

While recovery on various outcome measures occurs spontaneously after injury, much of this recovery may be due to behavioral compensation (Whishaw et al., 1991). For example, it is well-known in human stroke that compensatory movements of the trunk are employed during reaching (Cirstea and Levin, 2000). In the case of the study above, the combination of the increased disuse of the impaired digits, with the increase use of proximal could explain shifts in map topography. In a rat model of focal TBI, the rat equivalent of M1, the caudal forelimb area, was injured. In the absence of rehabilitative training (spontaneous recovery), behavioral performance on a pellet-reaching task improved over time, but at 5 weeks post-injury, and rats still have significant deficits (Nishibe et al., 2010). At this time point, the rat equivalent of premotor cortex (rostral forelimb area) contained a normal size forelimb representation. However, ICMS maps revealed a redistribution of forelimb representations: digit representations were reduced, while proximal representations were enlarged. Thus, at least in the absence of behavioral retraining, the plasticity in spared motor areas that occurs spontaneously may largely reflect the development of compensatory motor patterns, rather than true recovery of the original kinematic patterns (Figure 4).

The progression of recovery itself can be thought of a process of both reinstatement and relearning of lost functions, as well as adaptation and compensation of spared, residual function. Thus, it follows that the neurophysiological mechanisms that support learning in the intact cortex should mediate motor relearning and adaptation in the injured brain. Numerous studies over the last century and a half have provided substantial evidence to support the role of neural plasticity in functional recovery, both spontaneous and directed.

### Plasticity in adjacent, intact motor cortex after focal injury

Direct evidence that adjacent regions of the cortex might function in a vicarious manner after injury can be traced to studies in the mid-20th century (Glees and Cole, 1949). Monkeys were subjected to focal injury to the thumb representation. When brains were remapped following behavioral recovery, the thumb area reappeared in the adjacent cortical territory. However, using ICMS techniques, somewhat different findings were observed by Nudo et al. in the 1990s. Small, subtotal lesions were made in a portion of the distal forelimb representation (DFL) in squirrel monkeys, and the animals were allowed to recover spontaneously (i.e., without the benefit of rehabilitative training) for several weeks. In contrast to earlier finding, the remaining DFL was reduced in size, giving way to expanded proximal representations (Nudo and Milliken, 1996). However, in animals that underwent rehabilitative training with the impaired limb, the DFL was preserved or expanded (Nudo et al., 1996b). In retrospect, it is quite possible that the re-emergence of thumb representations in the early study by Glees and Cole may have been driven by post-injury behavioral demands.

Post-ischemia reorganization of sensorimotor cortex has also been demonstrated recently in transgenic mice expressing the light-sensitive channelrhodopsin-2 in layer V pyramidal neurons (Harrison et al., 2013). As in other studies, the neuronal activation in the peri-infarct cortex was reduced (Jones et al., 2013). Motor cortex infarcts resulted in the somatosensory map to emerge in the undamaged motor cortex near the lesion. This study adds additional credence to the notion that uninjured regions can play a vicarious function.

Studies in human stroke survivors also suggest that the intact, peri-infarct cortex may play a role in neurological recovery (Cramer et al., 1997; Jaillard et al., 2005; Teasell et al., 2005). Using transcranial magnetic stimulation (TMS) after stroke, it has been shown that the excitability of motor cortex is reduced near the injury, and the cortical representation of the affected muscles is decreased (Traversa et al., 1997; Butefisch et al., 2006). It is likely that this effect occurs from a combination of diaschisis-like phenomena and disuse of the affected limb (Liepert et al., 2000). Further, after several weeks of rehabilitation, motor representations in the injured hemisphere are enlarged relative to the initial post-injury map (Carey et al., 2002). Also, when goal-directed movement with the impaired hand is encouraged, a significant enlargement of the representation of the paretic limb is produced (Liepert et al., 1998), closely paralleling results in non-human primates.

Neuroanatomical changes occur in the peri-infarct cortex. Between 3 and 14 days post infarct, rats demonstrate increased GAP-43 immunoreactivity, suggesting significant neurite outgrowth in the peri-infarct region (Stroemer et al., 1995). Then, 14–60 days post-infarct, synaptophysin staining is elevated, signifying increased synaptogenesis. Local sprouting occurs in the peri-infarct area (Carmichael, 2006). Arteriolar collateral growth and new capillaries also form in the ischemic border (Wei et al., 2001). The picture is now emerging of an evolving peri-infarct environment in which growth inhibition is suppressed for about 1 mo post-infarct. This period is followed by “waves” of growth promotion which may modulate the brain's self-repair processes.

**Figure 5:**
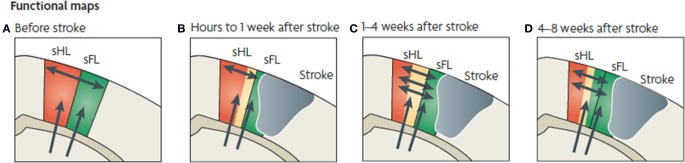
**Functional map changes in forelimb (sFL) and hindlimb (sHL) somatosensory cortex after a focal infarct in mouse**. Thalamic projections (arrows) and intracortical connections (double arrows) are also shown. **(A)** Normal somatosensory representation of sFL and sHL. **(B)** Within hours after focal infarct (gray), yellow areas show reduced sensory specificity, responding to both FL and HL stimulation. **(C)** Over the ensuing weeks, growth-promoting processes are triggered. Local axonal sprouting (double-headed arrows), dendritic spine expansion, and synaptogenesis occurs in the peri-infarct cortex. **(D)** Several weeks after stroke, specificity in sensory responses returns. Neurons that were formerly responsive to stimulation of hindlimb become responsive to forelimb stimulation (Murphy and Corbett, 2009).

In the past decade, several new techniques have been applied in animal models of ischemia that are giving us a vivid new insight into the temporal dynamics of post-injury plasticity (Sigler and Murphy, 2010). In an elegant series of experiments, Murphy and colleagues used two-photon microscopy in live animals in the mouse somatosensory cortex during ischemia caused by either injection of endothelin-1 (a potent vasoconstrictor) directly into the cortex, or by venous injection of Rose Bengal followed by photoactivation (photothrombotic stroke model) in the cortex (Brown and Murphy, 2008). When local ischemia was severe, spines were lost in less than 10 min. Dendritic spine loss is independent of NMDA receptor activation (Murphy et al., 2008). However, mitochondrial depolarization that might lead to cell death occurs within 1–3 min (Liu and Murphy, 2009). Dendritic spine loss and mitochondrial depolarization were reversible if reperfusion occurred within 1 h, and spines were restored (Zhang et al., 2005; Liu and Murphy, 2009). Sensory-evoked hemodynamic responses obtained using intrinsic optical imaging showed that sensory responses were blocked in the region of damaged dendrites (Zhang and Murphy, 2007) (Figure 5). This group has also taken advantage of the recent advances in transgenic models that are now readily available. By employing the photothrombotic stroke model to transgenic mice whose layer V cortical neurons were tagged with yellow fluorescent protein, the dendritic structure could be examined in single layer V neurons (Enright et al., 2007). The additional use of the dextran, Texas Red, allowed the investigators to precisely determine the border of the ischemic core. The results confirmed the dendritic damage in the ischemic core found with two-photon imaging in live animals. However, dendritic damage was limited to only 300 μm around the ischemic border. Beyond this zone, dendrites were essentially intact. Therefore, at least in this photothrombosis model for producing ischemic infarct, a substantial substrate for structural and functional plasticity exists in the peri-infarct cortex.

These techniques transgenic mouse models have now demonstrated that dendrites are remarkably modifiable for several weeks after ischemia. A significant increase in dendritic spine formation continues peaks at 1–2 weeks post-ischemia and persists for up to 6 weeks (Brown et al., 2007). During this time frame, dendritic arbors near the stroke shorten, while those away from the stroke extend (Brown et al., 2010). Studies by Carmichael and colleagues have shown in a mouse photothrombotic stroke model that functional reorganization takes place in the same peri-infarct region as does axonal sprouting, suggesting an anatomical substrate for functional plasticity (Clarkson et al., 2013).

### Functional and structural plasticity in remote regions after focal damage to M1

Primate brains are endowed with a rich intracortical network that allows reciprocal communication among the various sensory and motor areas. Injury to the motor cortex results in a potent disruption of integrated sensorimotor networks, resulting in loss of fine motor control. Upregulation of NMDA receptors and downregulation of GABA_A_ receptors occurs in the ipsilesional and contralesional hemisphere (Redecker et al., 2000). Remote cortical neurons that project to the ischemic core express genes related to axonal growth and guidance, dendritic growth and branching and cytoskeletal organization (Urban et al., 2012). It follows that disruption of the cortical motor network triggers a major reassembly of inter- and intra-areal cortical networks.

Since the development of compensatory behaviors and involvement of uninjured M1 are thought to contribute to spontaneous recovery, it follows that intact, motor areas outside of M1 may also contribute to recovery. Thus, it is plausible that following an injury to M1, the remaining, intact motor areas provide some role in functional recovery, via intracortical connectivity with other cortical regions and/or their direct corticospinal projection pathways.

Experiments by Liu and Rouiller (1999) showed in non-human primates that inactivation of premotor cortex with the GABAergic agonist muscimol following an M1 ischemic lesion reinstated behavioral deficits. This reinstatement was not observed with inactivation of the peri-lesional, or contralateral cortex. Thus, it follows that if premotor cortex is capable of compensating for the loss of motor function following an M1 injury, there should exist physiological changes that accompany this recovery. In adult squirrel monkeys ICMS mapping techniques were used to characterize representational maps of both M1 and PMv, before and after experimental ischemic infarcts that destroyed at least 50% of the M1 hand representation (Frost et al., 2003). All subjects showed an increased hand representation in PMv, specifically in digit, wrist, and forearm sites. Further, the amount of PMv expansion was correlated with the amount of the M1 hand representation that was destroyed. In other words, the more complete the M1 hand area lesion, the greater the compensatory reorganization in PMv. TMS studies in stroke survivors also suggests that premotor areas may serve in a vicarious capacity after injury (Fridman et al., 2004).

Interestingly, when lesions in the monkey models were smaller than 50% of the M1 hand area, the PMv hand representation decreased in size. Thus, examining the entire spectrum of M1 infarcts of varying sizes, the linear relationship is maintained. This result occurred despite the fact that some of these subtotal M1 hand area lesions nonetheless destroyed nearly the entire terminal field of PMv-M1 connections. What possible compensatory changes in the neuronal network could account for proportional gains in premotor hand areas, but losses with very small lesions? This phenomenon is reminiscent of Lashley's classic description of the relationship between cerebral mass and behavioral change (Lashley, 1930). According to this hypothesis, lesion size is generally assumed to be associated with the severity of deficits, while lesion location is related to the specificity of deficits. Lashley also proposed the concept of equipotentiality suggesting that each portion of a given cortical area is able to encode or produce behavior normally controlled by the entire area. In that vein, after smaller lesions, the surviving M1 tissue could potentially subserve the recovery of function. In that case, reorganization in distant, interconnected cortical areas would be a more “*passive*” process resulting from the loss of intracortical connections. This reorganization could be compared to a “sustained diaschisis” of PMv. After larger lesions, reorganization of the adjacent tissue may not suffice for normal motor execution. Thus, learning-associated reorganization would need to take place elsewhere, resulting in greater PMv expansion. Accordingly, in rats, the contralesional cortex is thought to be involved in behavioral recovery only after large lesions (Biernaskie et al., 2005). Interhemispheric signal processing occurs very rapidly after stroke, so that sensory responses produced by stimulation of either the contralateral or ipsilateral pathways are enhanced in the intact, contralesional cortex within 1 h. This disinhibition could not be explained simply by a transcallosal process (Mohajerani et al., 2011). While clearly there are short-term changes, later neuroanatomical changes in the intact cortex also occur as a use-dependent or skill-dependent change due to increased use of the less-affected limb (Bury and Jones, 2004).

Since neuroanatomical changes are known to occur in the peri-infarct area, and neuronal networks are densely interconnected, it follows that many of the functional changes that have been observed in cortical remote regions may have structural correlates. Evidence now exists that cortical efferent fibers are alterable in adults after cortical injury. After cortical lesions in rats, corticostriatal fibers, which primarily connect various cortical motor areas with the ipsilateral striatum, sprout from the intact (contralesional) cortex, and terminate in the contralateral striatum (i.e., on the side of the lesion) (Napieralski et al., 1996). Such plasticity in crossed corticofugal fiber systems may provide one mechanism for the remaining intact hemisphere to participate in recovery.

**Figure 6:**
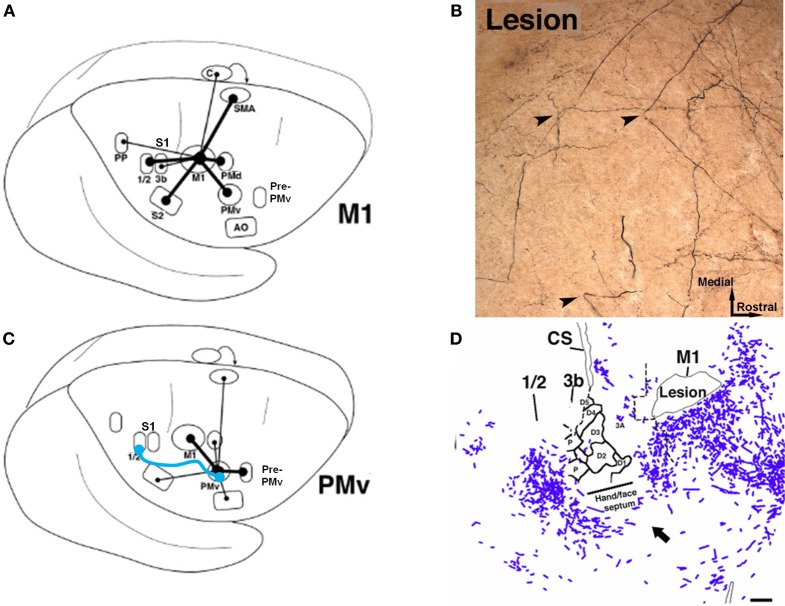
**Rewiring of corticocortical connections after ischemic infarct. (A)** In normal healthy squirrel monkeys, the primary motor cortex (M1) has dense reciprocal connections with both the premotor cortex (PMv, PMd, SMA) as well as the primary somatosensory cortex (S1) and the second somatosensory area (S2). **(B)** In addition to M1, the ventral premotor cortex (PMv) has dense connections with a rostral area called pre-PMv. PMv has moderate connections with S2, but negligible connections with S1. **(C)** Several weeks after an ischemic infarct in M1, axons originating in PMv can be seen making sharp bends and avoiding the infarct area, as shown in this tract-tracing study. **(D)** A low-magnification plot of axons within the section show that the axons originating from PMv course around the central sulcus. Substantial terminal bouton labeling (not shown) appears in S1 (areas 1 and 2). The blue line in **(B)** signifies the *de novo* pathway that forms after the lesion (Dancause et al., 2005).

Evidence that synaptogenesis and axonal sprouting occurs in the peri-infarct zone after a cortical injury was discussed above. Further, after an ischemic injury to the M1 hand representation in non-human primates, most intracortical connection patterns of the PMv remained intact (Dancause et al., 2005). However, when compared to uninjured control monkeys, after M1 lesions monkeys showed a remarkable proliferation of novel PMv terminal projections in primary sensory cortex (S1), specifically in the hand representations of areas 1 and 2 (Figure 6). Likewise, this somatosensory area had a significant increase in the number of retrogradely labeled cell bodies, indicating an increase in reciprocal projections from S1 to PMv. In addition, intracortical axonal projections from PMv significantly altered their trajectory near the site of the lesion. This finding is particularly interesting, given the direct intracortical connections between M1 and somatosensory cortex, as well as the presence of direct corticospinal projections originating from PMv. One hypothesis is that the post-injury sprouting represents a repair strategy of the sensorimotor cortex to re-engage the motor areas with somatosensory areas.

In intact brains, M1 receives input from various regions of the parietal lobe that supply cutaneous and proprioceptive information that is largely segregated in the M1 hand area—cutaneous information arriving in the posterior portion of M1, and proprioceptive information arriving in the more anterior portion. The functional importance of this somatosensory input can be appreciated from studies employing discrete lesions in these subregions in M1. Lesions in the posterior M1 hand area lesions result in behavioral deficits akin to those seen after S1 lesions. These deficits appear to be similar to sensory agnosia, in which the animal reaches for food items, but does not appear to know whether the item is actually in the hand. In contrast, anterior M1 hand area lesions result in deficits in metrics of the reach, perhaps indicating the disruption of proprioceptive information in the motor cortex (Friel et al., 2005). One lesson from these studies is that the motor cortex cannot be considered solely as a motor structure. Deficits result from sensory-motor disconnection in addition to disruption of motor output. Thus, after M1 injury, there is a substantial reduction of somatosensory input to motor areas. Perhaps, the novel connection between PMv and S1 is an attempt by the cortical motor systems to reconnect with somatosensory input. However, it is not yet known if this connection is functional, or if it is, whether it is adaptive or maladaptive. An alternate hypothesis is that the new pathway represents an aberrant connection that interferes with behavioral recovery.

It is likely that this phenomenon of intracortical sprouting of remote pathways interconnected with the injured zone is not a unique event. It is more likely that many structures, both cortical and subcortical, that are normally connected with the injured tissue undergo substantial physiological, and anatomical aterations. For instance, each of the other cortical motor areas (PMd, SMA, cingulate motor areas) are likely to change their intracortical connectivity patterns since their targets are destroyed. If so, it follows that the brain with a focal injury is a very different system. It is not simply a normal system with a missing piece. If intracortical reorganization is a predictable process, as we think it is, then we may be able to begin to develop ways of enhancing adaptive, while suppressing maladaptive connection patterns.

After stroke in humans, widespread changes occur in activation patterns, associated with movement of the paretic limb, in both the ipsilesional and contralesional hemispheres (Chollet et al., 1991; Weiller et al., 1993; Nelles et al., 1999). Whether such bilateral activation is adaptive or maladaptive is still a matter of debate, but it appears that as recovery proceeds, activation of the various regions in the ipsilesional cortex increases (Nelles et al., 1999; Carey et al., 2002). Increased ipsilateral activation after stroke is quite widespread, including spared premotor areas (Weiller et al., 1992; Seitz et al., 2005). In one longitudinal study, increased activation of SMA was correlated with better recovery (Loubinoux et al., 2003). Stroke survivors with MCA strokes that included lateral PM areas had poorer recovery (Miyai et al., 1999), while increased lateral PM activity was associated with better recovery (Miyai et al., 2003). In an experiment analogous to monkey secondary inactivation studies, the ipsilesional PMd of human stroke survivors was inactivated temporarily with repetitive TMS. This procedure resulted in reaction time delays that were not generated by inactivation of the contralesional PMd or the PMd of healthy subjects (Fridman et al., 2004). From the results to date, it is not possible to determine if any one motor area is more important in the recovery of motor abilities after stroke. We hypothesize that the entire cortical and subcortical motor system that is spared by the injury participates to varying degrees depending upon the extent and location of the injury and behavioral demands. At least some of the functions of the injured region(s) are thus redistributed across the remaining cortical and subcortical motor network.

## Interactions of injury and experience: neural correlates of learned non-use

In the acute period immediately after neural injury, motor ability is often severely impaired. Taub and colleagues have suggested that during this stage, attempts to complete tasks with the impaired limb are unsuccessful, or effortful, both of which are conditions that punish the use of the more-affected extremity, making future attempts less likely (Taub et al., 1998). By counter-conditioning this so-called “learned non-use” that develops in the acute phase permits latent motor abilities to be expressed. From this assumption, it was stipulated that function could be improved in chronic stroke patients. In particular, this group developed an innovative approach known as constraint-induced movement therapy (CIMT). The idea behind the application of CIMT originates from fundamental experiments conducted in non-human primates following peripheral deafferentation. In these experiments, disuse of the affected upper limb was observed following the injury. This maladaptive behavior persisted if no manipulation was introduced, even after a 3–6 month spontaneous recovery period. At that point, the function of the deafferented limb could be greatly enhanced by forcing its use by restraining the non-affected limb (Knapp et al., 1963). This led to the “learned non-use” hypothesis which stipulates that non-use, or less than maximal use, of the deafferented limb results from punishment of attempts to use the affected limb. This negative feedback would consist of unsuccessful behavioral consequences of attempts to use the affected upper limb (e.g., absence of reward for goal-directed activity, or painful execution). After the initial recovery period, when the ability to use the affected limb is stable, behavioral sequelae caused by the learned non-use remain. Therefore, because of the phenomenon, the actual use of the affected limb is much less than its true potential.

Strong support for the learned non-use formulation came from a study where restraint was applied directly following the deafferentation of the upper limb of an animal for a 3-month duration, therefore preventing the learned non-use phenomenon to occur When the restraint was removed, the animals used their deafferented limb (Taub, 1977). In the ensuing years, the learned disuse/nonuse model was employed in the CIMT approach. A landmark Phase III clinical trial has demonstrated that stroke survivors experience significant gains in functional outcomes after CIMT even years after stroke (Wolf et al., 2006).

The procedures used for CIMT are strikingly similar to the techniques used by Nudo and colleagues to demonstrate reorganization of the peri-infarct cortex following a focal stroke in non-human primates (Nudo et al., 1996b). After stroke, monkeys in the rehabilitative training group wore a jacket with a long sleeve that extended to a closed mitt on the less-impaired distal forelimb. This was necessary simply to test motor skill in the monkeys, since, without the restraint of the impaired limb, the monkey would simply switch to the less-impaired limb. However, if disuse (or nonuse) occurs after brain injury, there is a potential confound in the interpretation of post-injury plasticity. Behavioral experience and neural injury interact, such that changes in spared cortex are a product of both injury-related mechanisms, and behaviorally-driven changes, such as disuse (Woodlee and Schallert, 2004). How much of the change in motor maps is due to the injury alone vs. the disuse that follows the injury? If motor map integrity is powerfully modulated by motor experience, then the lack of experience should result in similarly large changes in map organization.

**Figure 7:**
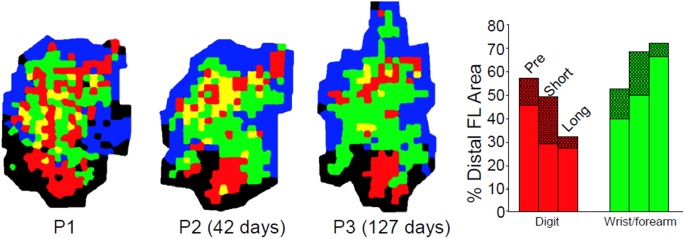
**Effects of disuse on motor maps in the absence of injury**. The preferred forelimbs of normal, healthy adult squirrel monkeys were placed in soft, restrictive casts for periods up to 5 months. ICMS mapping studies showed a progressive decrease in digit representation and a progressive increase in wrist/forearm representation. These effects were reversible after removal of the cast. These studies demonstrate that disuse has a substantial impact on motor cortex representations independent of the injury-induced disuse and neuropathological changes associated with stroke or traumatic injury (Milliken et al., 2013).

Disuse independent of neural injury has rarely been examined using mapping techniques as described either in humans or non-human primates. While some human TMS studies exist following casting, these are typically performed on individuals with fractures (Zanette et al., 2004). In a unique longitudinal study, Milliken et al. examined the organization of motor cortex in otherwise normal, healthy squirrel monkeys. Detailed ICMS maps were derived longitudinally, before and up to 35 weeks after restriction of the preferred distal forelimb in a soft cast (Milliken et al., 2013). The casted forelimbs were occasionally used for support, but were not able to be used for skilled movements. The results demonstrated a progressive re-distribution of digit and wrist/forearm representations in the M1 hand area. Digit area contracted, while wrist/forearm representations expanded (Figure 7). Furthermore, the changes were reversible After removal of the cast, behavioral skill generally returned within 1 week, and post-recovery maps returned to normal. Thus, disuse has neurophysiological consequences independent of the injury. However, as the effects of disuse in intact animals can be reversed, similarly, disuse after injury can also be reversed, at least if there is sufficient neural apparatus to support the execution of the motor task.

With respect to the integrity of the damaged hemisphere, the maladaptive effects go beyond simple disuse. Jones and colleagues have demonstrated in rat and mouse models that compensatory skill-learning by the less-affected limb impedes later functional recovery in the more-affected limb (Allred et al., 2005; Allred and Jones, 2008; Jones et al., 2013; Kerr et al., 2013). These maladaptive effects were not seen in animals that were trained on a bimanual task (Kerr et al., 2013).

## Windows of opportunity following brain injury based on neuroplasticity principles

As discussed above, following injury to the brain via trauma or stroke, a cascade of molecular and cellular events is set into motion in the surrounding tissue that results in both temporary and permanent changes in the anatomy and physiology of the affected structures. Many of these changes are pathological consequences of the injury (e.g., edema) and have potentially damaging results. However, many adaptive processes may begin early in the post-injury stage and result in reduction of pathophysiological events or in neuroplastic changes leading to at least some restoration of function (Witte and Stoll, 1997; Cramer, 2000). While a thorough understanding of these processes at the molecular, cellular and network levels is just beginning, sufficient knowledge is now available to begin testing hypotheses about the effects of specific post-injury interventions on functional recovery and its underlying neuroanatomical and neurophysiological bases.

The latent potential for enhancing neuroanatomical plasticity mechanisms after stroke has been demonstrated by the use of mutant mouse strains that lack the Nogo receptor. Nogo is a protein involved in the inhibition of axonal growth. Mice lacking the Nogo receptor recover motor function after stroke better than controls. Further, rats subjected to anti-No-go antibody treatment initiated 1 week after stroke resulted in better behavioral recovery compared with controls. Further, sprouting of contralateral corticorubral and ipsilateral corticospinal fibers was observed (Lee et al., 2004). Pharmacologic treatment with D-amphetamine after stroke has also been shown to enhance neocortical sprouting, synaptogenesis, and behavioral recovery after stroke in rats (Stroemer et al., 1998).

As a result of the abundance of evidence that has demonstrated that the brain is plastic after neuronal injury, and that behavioral experience can alter neuronal structure and function in both healthy and injured brains, it is now clear that principles of neuroplasticity can form the foundation for a wide range of therapeutic approaches to recovery. However, to develop effective, evidence-based rehabilitation protocols to promote recovery, two basic issues, timing and dose, still need to be addressed at molecular, cellular, and network levels of analysis.

Like many drug-based approaches to brain injury, there is most likely an optimal time period during which behavioral training paradigms are most effective. Upregulation of proteins involved in neural growth and guidance, many of which mimic events during neural development, occur over a relatively narrow window of time after injury. While recent clinical trials have demonstrated that outcome measures can be improved even years after stroke, the most optimal time may be during the period of maximum reorganization induced by the injury. Axonal sprouting takes place during a programmed process that is triggered, at least in animal models, by 1–3 days after stroke, and is fully mature by the end of 1 month (Carmichael, 2006). Neuronal growth-promoting and growth-inhibiting genes are turned on and off during similar post-injury time periods. In addition, events that trigger neurogenesis occur over a limited time period. Therefore, there is a critical need to understand how behavioral experience alters these reorganizational mechanisms differentially over time.

Likewise, any deleterious effects of behavioral interventions that are introduced too early in the process need further elaboration. Investigators and therapists in the field of neurorehabilitation became sensitized to this issue with the report in rats that early casting of the less-impaired limb resulted in exaggeration of neuronal injury (Kozlowski et al., 1996). The proposed mechanism for this use-dependent potentiation of the injury is that NMDA receptor-mediated processes are increased after brain injury, and that extreme overuse of the impaired limb causes further enhancement of these processes, ultimately resulting in glutamate excitotoxicity (Humm et al., 1999). Other studies have demonstrated smaller infarct volume and improved recovery in rats with MCA occlusion followed by treadmill running for 28 days (Matsuda et al., 2011). The authors also found increases in neurotrophic factors and decreases in apoptotic factors in the treadmill group. Thus, it is possible that the type and amount of motor activity may be critical in modulating the neural environment, and in determining whether regenerative processes or neuronal death cascades predominate during the early stages following brain injury. However, it should be noted that since rats are quadrupeds, the casting of the less-impaired limb constitutes a relatively severe form of overuse. The rat must use the impaired limb for postural support, grooming, feeding and locomotion. Whether this severe overuse phenomenon could occur simply via intense, repetitive training is not completely known.

These studies of early use raise the second issue of optimal “dose” of the behavioral experience. This factor is important not only for understanding the margin of safety for acute rehabilitation, but also the dose-response relationship for rehabilitation protocols across the continuum of the post-stroke intervention period. Animal models of experience-dependent recovery after injury have the advantage of utilizing highly motivated subjects that are on controlled feeding schedules. Thus, it has been common to implement protocols with very high levels of repetition compared with the equivalent therapy in human studies (Birkenmeier et al., 2010). Thus, it is important to define what human stroke patients can tolerate and whether more is better. A recent randomized controlled trial in 18 chronic stroke survivors demonstrated that doubling the number of repetitions in an upper-limb robot-assisted therapeutic intervention resulted in significant improvement in motor function (Hsieh et al., 2011). A recent meta-analysis also found limited evidence for a dose-response relationship in effecting motor recovery after stroke (Cooke et al., 2010). However, meta-analyses to examine dose-response relationships are complicated by the differences in how dose is defined (number of repetitions, number of days, number of sessions), and the various outcome measures that are employed. Studies that directly assess these relationships in both the acute and chronic stages after stroke are critically needed.

A rational, mechanistically-based approach to dose-response relationships can only be fully realized with analogous non-human animal studies to determine what molecular and cellular events are driven by increased dose. Does greater repetition simply result in increased neurotrophic factors? Is rehabilitation intensity related to greater synaptic number? At what point do these processes saturate? While ultimately, results of clinical trials, practicality and economics will drive our decisions regarding dose, developing a neurobiological model for this important factor will define the rules that govern limitations of therapy.

### Conflict of interest statement

The author declares that the research was conducted in the absence of any commercial or financial relationships that could be construed as a potential conflict of interest.
